# Bioimaging and Sensing Thiols In Vivo and in Tumor Tissues Based on a Near-Infrared Fluorescent Probe with Large Stokes Shift

**DOI:** 10.3390/molecules28155702

**Published:** 2023-07-27

**Authors:** Chunhui Ma, Dongling Yan, Peng Hou, Xiangbao Liu, Hao Wang, Chunhui Xia, Gang Li, Song Chen

**Affiliations:** 1 College of Pharmacy, Qiqihar Medical University, Qiqihar 161006, China; 2 Research Institute of Medicine & Pharmacy, Qiqihar Medical University, Qiqihar 161006, China

**Keywords:** near-infrared probe, thiols, bioimaging, tumor tissues

## Abstract

The well-known small-molecule biothiols have been used to maintain the normal metabolism of peroxy radicals, forming protein structures, resisting cell apoptosis, regulating metabolism, and protecting the homeostasis of cells in the organism. A large amount of research has found that abnormal levels of the above biothiols can cause some adverse diseases, such as changes in hair pigmentation, a slower growth rate, delayed response, excessive sleep and skin diseases. In order to further investigate the exact intracellular molecular mechanism of biothiols, it is imperative to explore effective strategies for real-time biothiol detection in living systems. In this work, a new near-infrared (NIR) emission fluorescence probe (probe 1) for sensitive and selective detection of biothiols was devised by combining dicyanoisophorone derivatives with the dinitrobenzenesulfonyl (DNBS) group. As expected, probe 1 could specifically detect biothiols (Cys, Hcy and GSH) through the dinitrobenzenesulfonyl group to form dye 2, which works as a signaling molecule for sensing biothiols in real samples. Surprisingly, probe 1 showed superior sensing characteristics and low-limit detection towards biothiols (36.0 nM for Cys, 39.0 nM for Hcy and 48.0 nM for GSH) with a large Stokes shift (134 nm). Additionally, the function of probe 1 as a platform for detecting biothiols was confirmed by confocal fluorescence imaging of biothiols in MCF-7 cells and zebrafish. More importantly, the capability of probe 1 in vivo has been further evaluated by imaging the overexpressed biothiols in tumor tissue. It is reasonable to believe that probe 1 can provide a valuable method to explore the relationship between biothiols and the genesis of tumor.

## 1. Introduction

As vital reactive sulfur species (RSS), intracellar thiols play a crucial role in regulating the redox balance of physiological and pathological processes [1,2]. The well-known small-molecule biothiols, including cysteine (Cys), homocysteine (Hcy) and glutathione (GSH), take on the mission of maintaining the normal metabolism of peroxy radicals, forming protein structures, resisting cell apoptosis, regulating metabolism, and protecting the homeostasis of cells in the organism [3,4]. A large amount of research has found that abnormal levels of the above biothiols (Cys, Hcy and GSH) can cause a series of related adverse diseases, such as changes in hair pigmentation, a slower growth rate, delayed response, excessive sleep, and skin diseases, and body obesity is closely consistent with the concentration fluctuations of Cys [5,6]. Excessive expression of Hcy in plasma is the main inducing factor of cardiovascular disease, and also results in degenerative lesions of cells in the nervous system [7,8]. In addition, as a indispensable endogenous antioxidant, the changes in GSH concentration are related to immune dysfunction, liver diseases, and many types of cancer [9]. Nowadays, it has been shown that these intrinsic biothiols can perform complicated biological roles in biological systems. There are still numerous confusing problems about the biological functional principles of biothiols to be solved [10]. Therefore, higher requirements have been put forward for tracing and evaluating biothiols in living cells or tissues. In order to further investigate the exact intracellular molecular mechanism of biothiols, it is imperative to explore effective strategies for real-time biothiol in living systems.

In recent decades, a number of methods have been used for the detection of biothiols, including capillary electrophoresis, chemiluminescence, high-performance liquid chromatography and colorimetric analysis [11,12,13]. Although these techniques can be well implemented for the analysis of biothiols, they have the drawbacks of complex sample handling, low sensitivity and high cost that restrict their utilization in biological samples. Nevertheless, fluorescent probes, as a convenience assay method, are considered advantageous for detecting target bioanalytes, allowing non-destructive detection, high selectivity, excellent spatial and temporal resolution and good biocompatibility [14,15,16,17,18]. Based on the above outstanding properties, fa luorescent probe has a wide range of applications in pharmacology, medicine, physiology and biology. In recent years, research on fluorescent probes has made phenomenal headway. A large number of fluorescent sensors for distinguishing biothiols over other amino acids have been reported, and are mainly based on different reactions, including cyclization of aldehydes, cleavage of sulfonamides, cleavage of disulfides, the Michael addition reaction and the intramolecular elimination reaction [19,20,21,22,23,24,25]. As far as we know, the vast majority of organic small-molecule fluorescence probes need to overcome a variety of defects in performance, such as strict test conditions, long response times, low sensitivity and selectivity, shorter-emission wavelengths, and high biotoxicity [26,27,28], which limit the further application of these fluorescent probes for the detection of various biomarkers in complex biological environments. Although there have been some advances in the development and practical application of biothiol probes, there is indeed a challenge in the alteration of short-emission wavelength, the small Stokes shift and the high detection limit. As a matter of fact, an enormous Stoke shift and near-infrared emission are vital preconditions for organic small-molecule fluorescent probes to be applied in vivo visualization [29,30,31]. Many fluorescent dyes have been used as fluorophores to synthesize and develop near-infrared fluorescent probes, including cyanine [32], BODIPY [33], methylene blue [34], and porphyrin [35]. Near-infrared emission probes with large Stokes shifts frequently have the ability to mitigate light scattering, undertake in-depth tissue imaging, and avoid self-quenching and autofluorescence. Therefore, it is indispensable to design a near-infrared emission fluorescent probe that has a large Stokes shift for biothiol detection in vitro and in vivo.

In this work, a new thiol-specific NIR fluorescent probe 1 was reported. The NIR luminophore dye 2 was devised by combining dicyanoisophorone with the 3′-formyl-4′-hydroxy-[1,1′-biphenyl]-4-carbonitrile to form a D-π-A structure. The dinitrobenzenesulfonyl (DNBS) group in probe 1 can not only quench fluorescence by the highly efficient PET process, but also serve as the functional trigger group for thiols. As expected, probe 1 displayed a fast response (<300 s), considerable specificity, a large Stokes shift (134 nm), good stability of pH, high sensitivity (36.0 nM for Cys, 39.0 nM for Hcy and 48.0 nM for GSH) and large NIR emission signal changes for biothiols. Furthermore, the excellent properties of probe 1 (including the Stokes shift, emission wavelength, detection limit and response time) were compared with previous literature (Table S1). Most importantly, bioimaging experiments in living MCF-7 cells, zebrafish and tumor tissues verified that probe 1 has potential as an effective tool for sensing biothiols in living systems.

## 2. Results and Discussion

### 2.1. Design and Synthesis of Probe 1

**Scheme 1:**
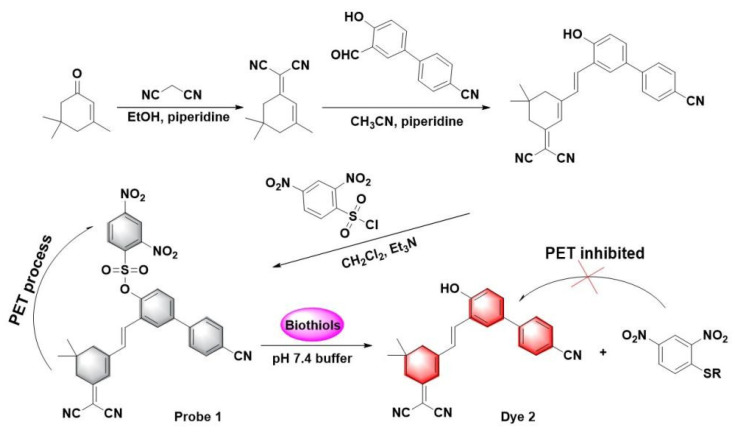
The synthesis and design strategy for probe 1.

The design strategy and recognition mechanism of probe 1 towards biothiols (Cys, Hcy and GSH) can be appropriately explained by Scheme 1. Due to their great features in near-infrared emission wavelengths and large Stokes shift, as an excellent fluorophore, dicyanoisophorone derivatives have been reported to exhibit fascinating properties in the design of small-molecule fluorescent probes [36,37]. Based on the previous research results, we rationally designed the dicyanoisophorone derivative (dye 2) with a long-wavelength emission which was located in the near-infrared emission region; meanwhile, we chose dinitrobenzenesulfonyl as the response group, and linked these two parts via an ester bond. Eventually, a biothiol-responsive near-infrared fluorescent probe (probe 1) was obtained. Novel fluorescent probe 1 was nearly non-fluorescent in the red region because of the PET process between the electron-donor dicyanoisophorone unit and the electron-acceptor dinitrobenzenesulfonyl group [38,39]. Upon response with biothiols, the dinitrobenzenesulfonyl group in probe 1 was easily attacked by biothiols (Cys, Hcy and GSH) based on a nucleophilic substitution reaction. Correspondingly, the mixing solution generated obvious fluorescence in the red region because of th PET process inhibition. The structural design of fluorescent probe 1 is supported by the spectroscopy results. HRMS analysis also provided the evidence for the reaction mechanism between probe 1 and the biothiols (Figure S12). These properties enabled probe 1 to be applied for the detection of biothiols with a near-infrared signal. Probe 1 was synthesized by a three-step continuous reaction, and the corresponding data (^13^C NMR, ^1^H NMR and HRMS) of probe 1 are given in the Supporting Information (Figures S6–S11).

### 2.2. Fluorescence Properties of Probe 1

**Figure 1:**
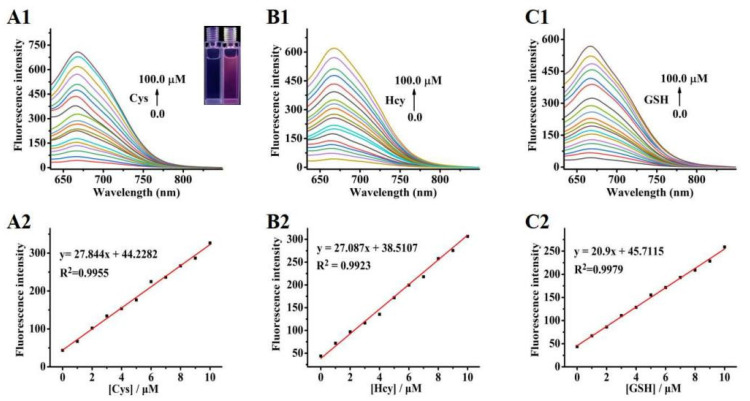
The fluorescent intensity change in probe 1 (10.0 μM) with an increase in biothiols (0.0–100.0 μM) ((**A1**) corresponds to Cys, (**B1**) corresponds to Hcy and (**C1**) corresponds to GSH); insert (**A1**): change in fluorescent color of probe 1 (10.0 μM) without and with 100.0 μM Cys). Linear correlation with emission intensity at 670 nm and an incremental concentration of (0.0–10.0 μM) biothiols ((**A2**) for Cys, (**B2**) for Hcy, and (**C2**) for GSH)).

Firstly, the optical response of probe 1 towards three common biothiols (Cys, Hcy and GSH) in pH 7.4 PBS (containing 1.0 mM CTAB) was investigated. As can be seen in Figure 1, probe 1 was almost non-emissive at 670 nm, which was attributed to photo-induced electron transfer (PET) effect of the dinitrobenzenesulfonyl group in probe 1. As expected, upon reacting with biothiols (0.0–100.0 μM), it was found that the fluorescence peak at 670 nm increased gradually (Figures S2–S4). Simultaneously, the fluorescence color changed from colorless to red under 365 nm ultraviolet light. The present of a large Stokes shift (134 nm) can eliminate the internal influence of the sample to empower probe 1 with superior sensitivity. What is more important is that the fluorescence intensity has a favorable linear relationship (y = 27.844x + 44.2282, R^2^ = 0.9955 for Cys, y = 27.087x + 38.5107, R^2^ = 0.9923 for Hcy and y = 20.9x + 45.7115, R2 = 0.9979 for GSH) with a low biothiol concentration (0.0–10.0 μM). Based on 3 σ/k (σ represents the standard deviation of three blank samples, and S represents the linear slope), the detection limit of probe 1 was determined to be 36.0 nM for Cys, 39.0 nM for Hcy and 48.0 nM for GSH. The overall results implied that probe 1 has excellent potential for quantitative biothiol detection in practical application.

### 2.3. The Selectivity of Probe 1

**Figure 2:**
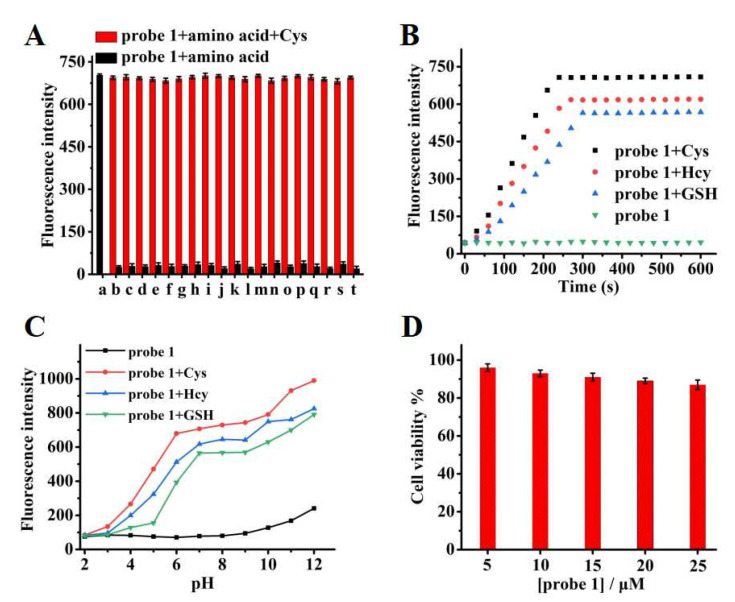
Study on the properties of probe 1. (**A**) Fluorescence intensity after a treatment with 100.0 μM single analyte (black columns) in comparison to after the addition of Cys (red columns) (a–t: Cys, Arg, Asn, Asp, His, Leu, Gly, Pro, Thr, Tyr, Val, Na^+^, Ca^2+^, Mg^2+^, Zn^2+^, SO_3_^2−^, CO_3_^2−^, SO_4_^2−^, NO_3_^−^, and PO_4_^3−^). (**B**) Time-dependent increase in the fluorescence of free probe 1 (10.0 μM) with biothiol (100.0 μM for Cys, Hcy and GSH). (**C**) Changes in the fluorescence intensity of probe 1 (10.0 μM) at different pH values in the absence and presence of (100.0 μM) biothiol (Cys, Hcy and GSH). (**D**) Percentage of viable MCF-7 cells after treatment with indicated concentrations of probe 1 (5.0, 10.0, 15.0, 20.0, and 25.0 μM) for 24 h.

Considering the extreme complexity of the biological internal environment, it is crucial to screen the specificity and anti-interference ability of probe 1 towards amino acids and ions (Figure 2). When probe 1 was added with different analytes, it was found that there was no obvious fluorescence in the presence of 100.0 μM analytes (including Arg, Asn, Asp, His, Leu, Gly, Pro, Thr, Tyr, Val, Na^+^, Ca^2+^, Mg^2+^, Zn^2+^, SO_3_^2−^, CO_3_^2−^, SO_4_^2−^, NO_3_^−^, and PO_4_^3−^), whereas there was an increase in fluorescence at 670 by the incubation of Cys (100.0 μM). Anti-interference by probe 1 for biothiols in the presence of coexisting substances was investigated. As depicted in Figure 2A, the effect of common interfering substances (such as various amino acids and inorganic species) on the behavior of probe 1 towards biothiols was almost negligible. The above results on the changes in fluorescence at 670 nm clearly indicated that probe 1 could detect biothiols with high selectivity even in a complex biological system.

### 2.4. Response Time and pH Study of Probe 1

In order to investigate the real-time detection ability of probe 1 in an actual sample, the fluorescence kinetic profiles of probe 1 with 100.0 μM biothiols (Cys, Hcy and GSH, respectively) at 670 nm were thus determined. As described in Figure 2B, probe 1 responded rapidly towards biothiols, as all the increase in fluorescence reached a stable level of less than 300 s (240 s for Cys, 270 s for Hcy and 300 s for GSH). In comparison, the minimal change in fluorescence intensity for free probe 1 could almost be neglected over the same assay time. Further, the effect of pH on the fluorescence response of probe 1 towards biothiols was further studied. Probe 1, in the absence of biothiols, exhibited excellent stability in an environment with a wide pH range of 2.0–9.0. After the addition of biothiols (100.0 μM for Cys, Hcy and GSH), a prominent change in fluorescence emerged across the rnage of pH 6.0–9.0 (Figure 2C). From this, it was confirmed that probe 1 could be used to rapidly monitor biothiols in physiological conditions.

### 2.5. Imaging of MCF-7 Cells

**Figure 3:**
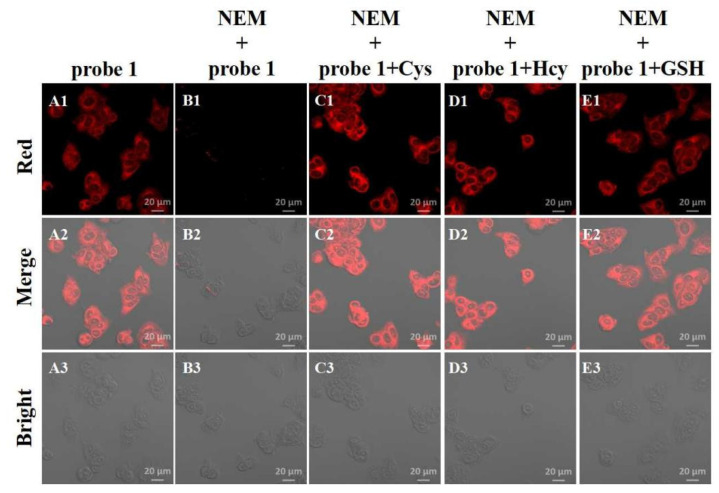
Confocal fluorescence images for biothiols in living MCF-7 cells. (**A1**–**A3**) MCF-7 cells only stained with free probe 1 (10.0 μM); (**B1**–**B3**) MCF-7 cells incubated with N-ethylmaleimide (NEM, 1.0 mM) followed by treatment with probe 1 (10.0 μM); (**C1**–**E3**) MCF-7 cells pre-incubated with N-ethylmaleimide (NEM, 1.0 mM) before incubation with probe 1 (10.0 μM), and then the living cells were treated with biothiols (Cys, Hcy and GSH).

Inspired by the sensing capacity of probe 1 towards biothiols (Cys, Hcy and GSH) in solution, the imaging performance of probe 1 towards intracellular biothiols was estimated next. Firstly, the cytotoxicity of probe 1 was evaluated by using the MTT assay. As displayed in Figure 2D, when MCF-7 cells were incubated with probe 1 at concentrations of 5.0, 10.0, 15.0, 20.0 and 25.0 μM for 24 h, the viability of MCF-7 cells was more than 95%. It is indicated that there was low cytotoxic effect of probe 1 in MCF-7 cells. Subsequently, the confocal fluorescence imaging of probe 1 towards biothiols was carried out in living MCF-7 cells. MCF-7 cells were added with probe 1 alone (10.0 μM), which showed a red fluorescent signal. After being pretreated with a thiol scavenger, N-ethylmaleimide (NEM, 1.0 mM), and stained with probe 1 (10.0 μM), the red fluorescent signal in MCF-7 cells disappeared. While the addition of the biothiols (100.0 μM for Cys/Hcy/GSH, respectively), a significant increase in fluorescence in the red emission channel was seen on the incubation of NEM cells (Figure 3). These red-channel imaging experiments confirmed that probe 1 could be used as a detector to visualize biothiols in living cells.

### 2.6. Fluorescence Imaging of Zebrafish

**Figure 4:**
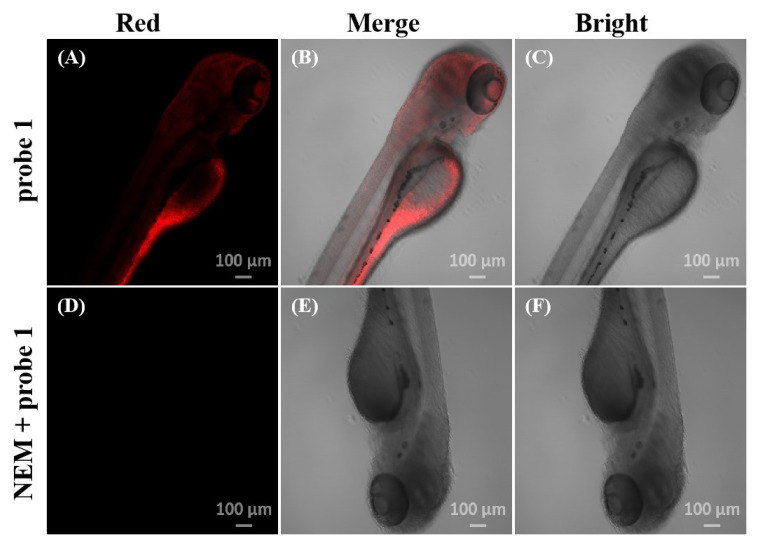
Confocal fluorescence images of living zebrafish. (**A**–**C**) Zebrafish were stained with probe 1 (10.0 μM); (**D**–**F**) Zebrafish were treated with N-ethylmaleimide (NEM, 1.0 mM) and stained with free probe 1 (10.0 μM).

It is well known that zebrafish is a suitable animal model for imaging in vivo. Here, zebrafish was arranged to evaluate the utility of probe 1 for biothiol detection. As illustrated in Figure 4, when zebrafish was fed with probe 1 (10.0 μM), a red fluorescent signal originating from the endogenous biothiols of zebrafish was observed. In contrast, after the effective inhibition of 1.0 mM NEM, the bright fluorescent signal disappeared in the red channel. These data indicated that probe 1 possessed good biothiol-sensing ability in zebrafish.

### 2.7. Confocal Imaging in Fresh Tissue

**Figure 5:**
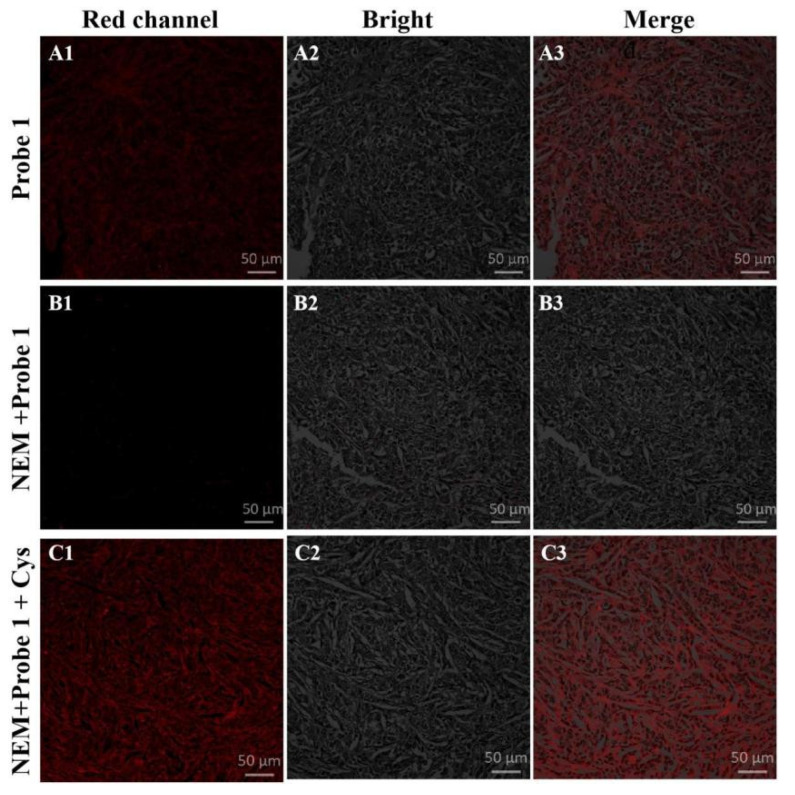
Fluorescence images of biothiols in tissues with probe 1. (**A1**–**A3**) were only loaded with probe 1 (50.0 μM), (**B1**–**B3**) were pre-treated with N-ethylmaleimide (NEM, 1.0 mM) and further incubated with probe 1 (50.0 μM), (**C1**–**C3**) were pre-stimulated with N-ethylmaleimide (NEM, 1.0 mM) prior to being co-incubated with probe 1 (50.0 μM) and Cys (200.0 μM).

Encouraged by the bio-sensing performance of probe 1 for biothiols in cells and zebrafish, we attempted to inspect the capability of biothiol detection in sw 1990 pancreatic cancer cells inoculated 20~25 g of nude mice. It was shown in Figure 5 that a red fluorescent signal could be traced when tissues were only loaded with 50.0 μM probe 1 for 1 h. Moreover, in the case of pre-treatment with NEM, the light in the red channel which stem from the reaction between probe 1 and endogenous biothiols was quenched. In comparison, the obvious fluorescence in the red channel was viewed by treatment of the NEM-tumor tissue sections with probe 1 (50.0 μM) and Cys (200.0 μM). These outcomes proved that probe 1 is a valuable molecular tool for biothiol identification in cancer diagnosis and research.

## 3. Materials and Methods

### 3.1. Materials and Instruments

All reagents used in the experiments were purchased from Sinopharm Chemical Reagent Co., Ltd. (Shanghai, China) and not further purified prior to use. UV–vis spectra were measured on a Shimadzu UV-2450 spectrophotometer. The emission spectra were recorded by a Shimadzu RF5301PC spectrophotometer. NMR spectra were collected on A BRUKER 400 NMR spectrometer. Mass spectrometry was fulfilled by a Waters ^®^ Xevo G2-S QTof™ mass spectrometer. Fluorescence imaging of living cells and zebrafish was performed on a Zeiss LSM710 microscope.

### 3.2. Spectral Measurements

The stock solution of probe 1 (1.0 mM) was properly dissolved in dimethyl sulfoxide (DMSO). The stock solutions (10.0 mM) of Cys, Hcy, GSH and other analytes (Arg, Asn, Asp, His, Leu, Gly, Pro, Thr, Tyr, Val, Na^+^, Ca^2+^, Mg^2+^, Zn^2+^, SO_3_^2−^, CO_3_^2−^, SO_4_^2−^, NO_3_^−^, and PO_4_^3−^) were prepared in pH 7.4 PBS (containing 1.0 mM CTAB) buffer solution and freshly used. The reaction solutions were comprised of probe 1 and various analytes, and measured after shaking for 300 s at room temperature. The 600 nm excitation wavelength and 10.0 nm/10.0 nm slit width were adopted on the spectroscopic measurement.

### 3.3. Synthesis of Dye 2

2-(3,5,5-trimethylcyclohex-2-en-1-ylidene)malononitrile (0.186 g, 1.0 mmol) and 3′-formyl-4′-hydroxy-[1,1′-biphenyl]-4-carbonitrile (0.223 g, 1.0 mmol) were added to ethanol (15.0 mL) with a moderate amount of piperidine. The mixture was refluxed at 85 °C under N2 for 6 h. The reactants were cooled to room temperature. The precipitated solid was washed three times by filtration with cold ethanol and dried to obtain product dye 2 as an orange solid (45.7%). ^1^H NMR (400 MHz, DMSO) δ 10.53 (s, 1H), 8.15 (s, 1H), 7.91 (d, *J* = 6.6 Hz, 4H), 7.57 (dt, *J* = 26.9, 11.9 Hz, 3H), 7.03 (d, *J* = 8.3 Hz, 1H), 6.89 (s, 1H), 2.62 (s, 2H), 2.50 (s, 2H), 1.03 (s, 6H). ^13^C NMR (100 MHz, DMSO) δ 170.83, 157.38, 156.71, 144.56, 133.16, 132.21, 130.01, 129.94, 129.88, 127.30, 126.23, 123.72, 123.01, 119.52, 117.32, 114.40, 113.63, 109.57, 76.47, 42.84, 39.81, 39.60, 39.39, 38.63, 32.17, and 27.89. HRMS (EI) *m*/*z* calcd for [C_26_H_21_N_3_O + H]^+^: 392.1763; found: 392.1743.

### 3.4. Synthesis of Probe 1

Dye 2 (0.04 g, 0.1 mmol), 30.0 μL of triethylamine and 2,4-dinitrobenzenesulfonyl chloride (0.035 g, 0.13 mmol) were mixed by 10.0 mL of dichloromethane. The reaction took place at room temperature for 2 h. The final solution was distilled, and then purified by column chromatography (petroleum ether/ethyl acetate = 4:1, *v*/*v*) to obtain probe 1 (75.6%). ^1^H NMR (400 MHz, DMSO) δ 9.19 (s, 1H), 8.61 (d, *J* = 6.3 Hz, 1H), 8.29 (s, 2H), 8.01 (d, *J* = 6.1 Hz, 4H), 7.88 (d, *J* = 6.8 Hz, 1H), 7.50 (d, *J* = 16.0 Hz, 2H), 7.07 (d, *J* = 16.1 Hz, 1H), 6.85 (s, 1H), 2.63 (s, 2H), 2.32 (s, 2H), 1.00 (s, 6H). ^13^C NMR (100 MHz, DMSO) δ 169.46, 153.45, 151.04, 147.52, 145.86, 142.01, 137.96, 133.04, 132.28, 130.13, 129.61, 128.81, 127.40, 126.99, 125.79, 123.84, 123.66, 120.21, 118.11, 112.90, 112.06, 110.23, 77.56, 41.48, 37.34, 30.98, and 26.69. HRMS (EI) *m*/*z* calcd for [C_32_H_23_N_5_O_7_S + H]^+^: 622.1396; found: 622.1347.

### 3.5. Cell Culture and Fluorescence Imaging

MCF-7 cells were placed in Dulbecco’s modified eagle medium (DMEM) containing 1% penicillin streptomycin and 10% FBS (fetal bovine serum) and incubated for 24 h at 5% CO_2_ and 37 °C in a humid environment to adhere. As control groups, some MCF-7 cells were treated with 10.0 μM probe 1 at 37 °C for 30 min, and some MCF-7 cells were treated with 1.0 mM N-ethylmaleimide (NEM, a biothiol scavenger) for 30 min at 37 °C, and stained by probe 1 (10.0 μM) for 30 min. As for the experimental group, MCF-7 cells were firstly treated with NEM (1.0 mM) for 30 min at 37 °C, and then stained with probe 1 (10.0 μM) for another 30 min. Finally, three experimental groups were added with Cys/Hcy/GSH (100.0 μM) for 30 min, respectively. Cell images were carried out using a confocal fluorescence microscope after washing with PBS buffer.

### 3.6. Zebrafish Imaging

Zebrafish were kept in an E2 embryo medium at 28 °C for light and dark cycle rearing. All the zebrafish were then studied using the protocols supported by the Animal Experimentation Ethics Care Committee of Qiqihar Medical University (QMU-AECC-2023-72). Imaging of endogenous biothiols in zebrafish was performed based on the coloration of 10.0 μM probe 1—alone for 30 min. Zebrafish of the control group were pre-incubated with NEM (1.0 mM) for 30 min, and then loaded in the probe 1 solution for another 30 min. Finally, the zebrafish were washed with PBS three times to remove residue and imaged in the red channel by a confocal fluorescence microscope.

### 3.7. Tissue Imaging

The Balb/c mice (female) were obtained from Liaoning Changsheng biotechnology Co., Ltd. (Benxi, China). The Balb/c mice were fed in a room at a temperature of 22 ± 2 °C and a humidity of 50 ± 10%. All mice were then studied using the protocols supported by the Animal Experimentation Ethics Care Committee of Qiqihar Medical University (QMU-AECC-2023-72). At the age of 4 weeks, Balb/c mice were randomly divided into three groups (groups A, B, and C). Immediately, the mice were injected with sw 1990 cancer cells in the armpit to establish the tumor model. For the endogenous thiol imaging experiments (groups A), the mice were injected with probe 1 (50.0 μM, 100.0 μL) through tail vein. After incubation of probe 1 for 1 h, the tumors were anesthetized and made into suitable tissue sections. For the exogenous thiol imaging experiments (groups C), the prepared tissue section was treated with NEM (1.0 mM) for 1 h and then incubated with probe 1 (50.0 μM) for 1 h. In addition, NEM-stained tissue sections (groups B) were treated with probe 1 (50.0 μM) for 1 h and Cys (200.0 μM) for another 1 h. Before imaging, all the tissue sections were washed three times with PBS.

## 4. Conclusions

In conclusion, we developed a new fluorescent probe for biothiol (Cys, Hcy and GSH) detection. Probe 1 can react with biothiols through a sulfhydryl-promoted nucleophilic substitution reaction to release a remarkably fluorescent dye. The analysis of the fluorescent spectra identified the reaction between probe 1 and the biothiols (Cys, Hcy and GSH). Because sulfhydryl promoted the nucleophilic substitution reaction and the PET process of the molecular structure, probe 1 enabled selective near-infrared fluorescent imaging of biothiols (Cys, Hcy and GSH) with great sensitivity (the limit of detection of probe 1 was calculated to be 36.0 nM for Cys, 39.0 nM for Hcy and 48.0 nM for GSH) and a quick response speed (240 s for Cys, 270 s for Hcy and 300 s for GSH). Probe 1 could be applied for detecting biothiols in physiological conditions (pH 7.4) and can be successfully used for imaging biothiols in living MCF-7 cells and zebrafish. Most importantly, probe 1 was used to monitor the upregulated biothiols in tumor sections. This is of great significance for revealing the role of biothiols in the complex pathological environment of tumors. Further, it is hoped that this study will be instructive in studying the mechanism of antitumor drugs. Therefore, we anticipate that probe 1 could serve as a superior fluorescent tool to facilitate biomedical research into the role of small-molecule biothiols (Cys, Hcy and GSH) in cancer cells.

## Data Availability

The data presented in this study are available in the Supplementary Materials.

## Supplementary Materials

The following supporting information can be downloaded at: https://www.mdpi.com/article/10.3390/molecules28155702/s1, Table S1. Comparison of fluorescent probes for thiols. Figure S1. UV–Vis spectral responses of dye 2 (black line), probe 1 (red line), probe 1 to Cys (purple line)/Hcy (pink line)/GSH (green line). Figure S2. Fluorescence intensity of probe 1 (10.0 μM). at 670 nm in response to different concentrations of Cys (0.0-100.0 μM). Figure S3. Fluorescence intensity of probe 1 (10.0 μM). at 670 nm in response to different concentrations of Hcy (0.0–100.0 μM). Figure S4. Fluorescence intensity of probe 1 (10.0 μM). at 670 nm in response to different concentrations of GSH (0.0–100.0 μM). Figure S5. Cell viability of MCF-7 cells after treatment with indicated concentrations of probe 1 after 24 h. Figure S6. ^1^H NMR spectrum of dye 2 in DMSO-*d_6_*. Figure S7. ^13^C NMR spectrum of dye 2 in DMSO-*d_6_*. Figure S8. Mass spectrum of dye 2. Figure S9. ^1^H NMR spectrum of probe 1 in DMSO-*d_6_*. Figure S10. ^13^C NMR spectrum of probe 1 in DMSO-*d_6_*. Figure S11. Mass spectrum of probe 1. Figure S12. Mass spectrum of probe 1 with Cys.

## Sample Availability

Samples of all compounds are available from the authors.
