# Supplementary material for: Bioimaging and Sensing Thiols In Vivo and in Tumor Tissues Based on a Near-Infrared Fluorescent Probe with Large Stokes Shift
Source: Molecules. 2023 Jul 27;28(15):5702. doi: 10.3390/molecules28155702 (PMC10419645; doi:10.3390/molecules28155702)
Supplement: Supplementary file 1 [file molecules-28-05702-s001.zip › molecules-2517402-supplementary.pdf]

Supplementary Materials

Bioimaging and Sensing Thiols In Vivo and in Tumor Tissues Based on a Near-Infrared Fluorescent Probe with Large Stokes Shift

Chunhui Ma [1], Dongling Yan [1], Peng Hou [1], Xiangbao Liu [1], Hao Wang [1], Chunhui Xia [1], Gang Li [2] and Song Chen [1,]*

[1] College of Pharmacy, Qiqihar Medical University, Qiqihar 161006, China
[2] Research Institute of Medicine & Pharmacy, Qiqihar Medical University, Qiqihar 161006, China
* Correspondence: chensong@qmu.edu.cn

Table of contents

Page

Table S1..........1

Figures S1-2..........4

Figures S3-4..........5

Figures S5-6..........6

Figures S7-8..........7

Figures S9-10..........8

Figures S11-12..........9

Table S1. Comparison of fluorescent probes for thiols.

Probe	Stokes shift	Emission	Detection limit	Response time	Applications	Literature
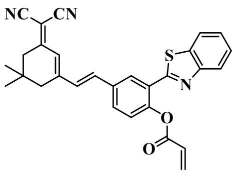	176nm	718nm	Cys (0.09μM)/<br>Hcy (0.12μM)	Cys (2min)/<br>Hcy (20min)	HeLa cell	Dyes and Pigments 2022,203, 110320
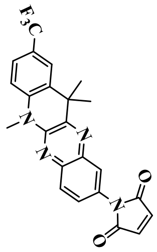	95nm	495nm	Cys (36.6nM)/<br>Hcy (116.5 nM)/<br>GSH (57.1 nM)	Cys (30min)/<br>Hcy (180min)/<br>GSH (180min)	HeLa cell<br>zebrafish	Tetrahedron 2023,139, 133433
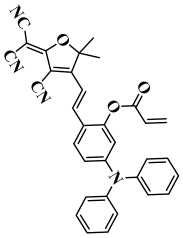	106nm	666 nm	Cys (73.0 nM)	Cys (40min)	HeLa cell	Tetrahedron 2023,140, 133492
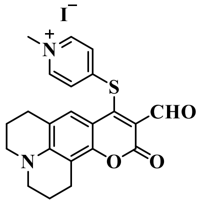	Cys/Hcy: 104nm<br>GSH: 61nm	Cys/Hcy: 504nm<br>GSH: 566nm	Cys (132nM)/<br>Hcy (105 nM)/<br>GSH (62 nM)	Cys (190s)/<br>Hcy (155s)/<br>GSH (80s)	HeLa cell<br>zebrafish	Dyes and Pigments 2022,199, 110058
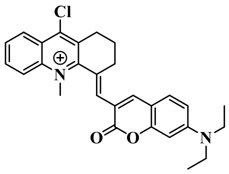	194nm	674nm	Cys (46nM)/<br>Hcy (83 nM)	Cys (3min)/<br>Hcy (15min)	A549 cell<br>Mice	Sensors and Actuators: B. Chemical 2023,374, 132799

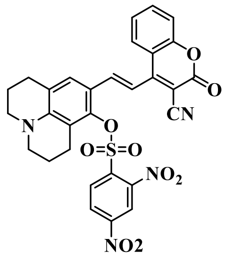	78nm	679nm	Cys (7.9nM)/ Hcy (10.2 nM)/ GSH (4.2 nM)	Cys (12min)/ Hcy (15min)/ GSH (9min)	A549 cell	Journal of Luminescence 2021,234, 117994
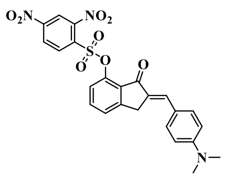	113nm	563nm	Cys (0.471μM)/ Hcy (0.638μM)/ GSH (0.384μM)	10–20 min	HeLa cell	Spectrochimica Acta Part A: Molecular and Biomolecular Spectroscopy 2023,300, 122870
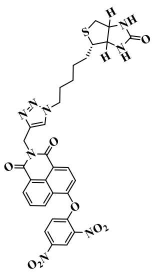	120nm	570nm	Cys (27n M)	/	HepG2 cell zebrafish	Journal of Photochemistry & Photobiology, A: Chemistry 2023,444, 114919
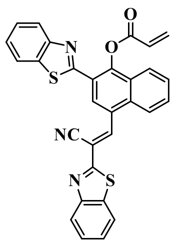	107nm	620nm	Cys (0.076μM)	Cys (15min)	PC3 cell Mice	Journal of Photochemistry & Photobiology, A: Chemistry 2023,436, 114383
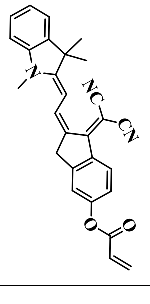	30nm	625nm	Cys (1.92μM)/ Hcy (3.46μM)	Cys/Hcy (20min)	HepG2 cell	Spectrochimica Acta Part A: 2022,279, 121364

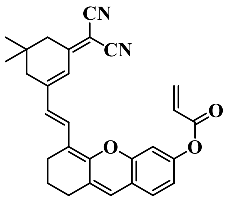	180nm	770nm	Cys (0.4μM)	Cys (10min)	HCT116 cell Mice	Analytica Chimica Acta 2021,1171, 338655
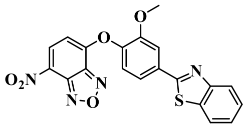	75nm	405nm	Cys (0.03μM)/ GSH (0.14μM)/ H_2S(0.15μM)	/	293 T cell Beer	Journal of Fluorescence 2022,32, 175-188
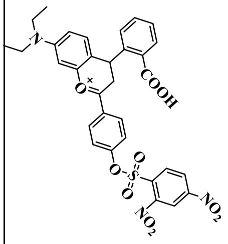	57nm	632nm	Cys (1.47μM)/ Hcy (2.4μM)/ GSH (2.27μM)	Cys (60min)/ Hcy (40min)/ GSH (60min)	HeLa cell	Journal of Photochemistry & Photobiology, A: Chemistry 2022,425, 113654
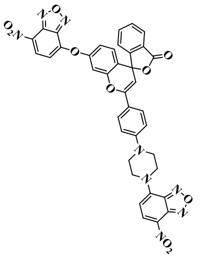	Cys:75nm H_2S： 58nm	Cys:553nm H_2S： 604nm	Cys (58.4nM)/ H_2S (81.1nM)	Cys (0.5h)/ H_2S (2.5h)	MCF-7 cell	Journal of Photochemistry & Photobiology, B: Biology 2022,230, 112441
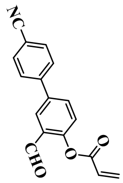	55nm	520nm	Cys (3.8 nM)	Cys (3min)	MCF-7 cell HL60 cell zebrafish	Spectrochimica Acta Part A: 2023,294, 122523
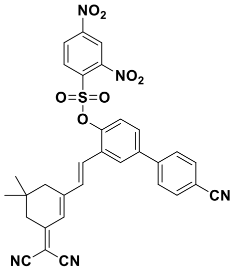	**134 nm**	**670 nm**	**Cys (36.0 nM)/ Hcy (39.0 nM)/ GSH (48.0 nM)**	**Cys (240 s)/ Hcy (270 s)/ GSH (300 s)**	**MCF-7 cell Zebrafish tumor tissues**	**This work**

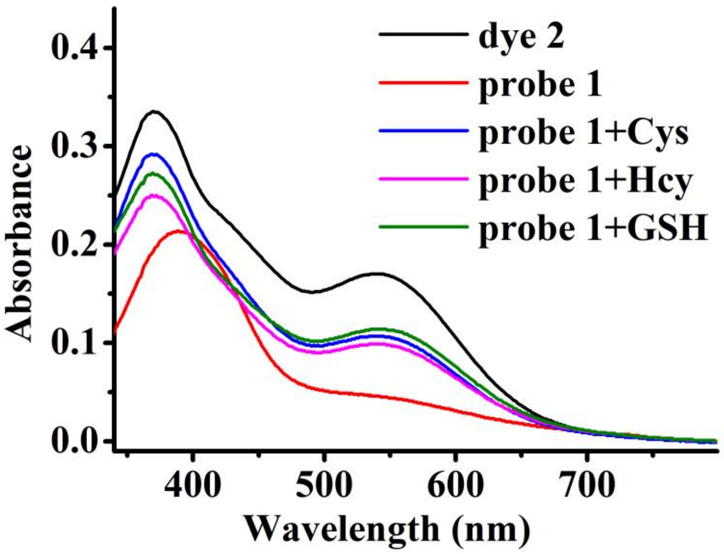

Figure S1. UV–Vis spectral responses of dye **2** (black line), probe **1** (red line), probe **1** to Cys (purple line) / Hcy (pink line) / GSH (green line).

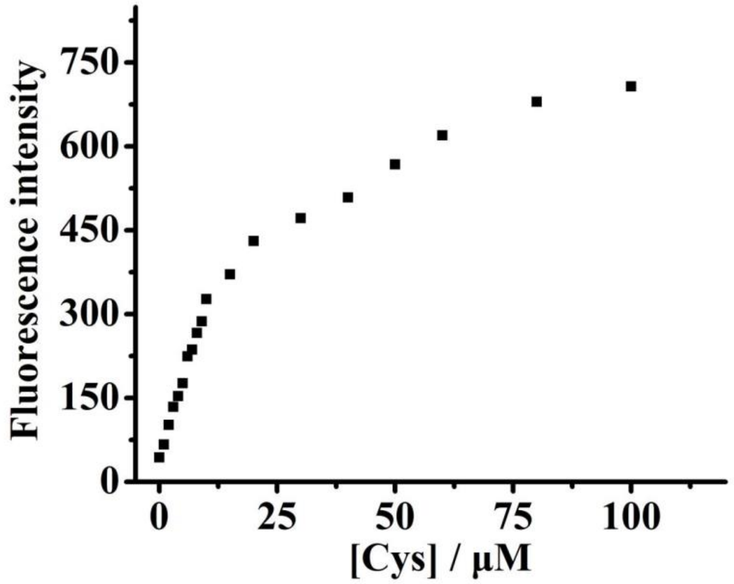

Figure S2. Fluorescence intensity of probe **1** (10.0 μM). at 670 nm in response to different concentrations of Cys (0.0-100.0 μM).

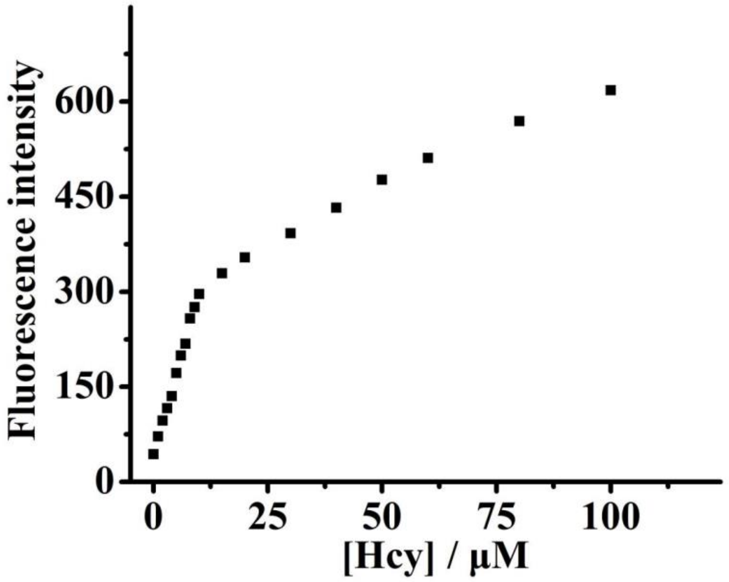

Figure S3. Fluorescence intensity of probe **1** (10.0 μM). at 670 nm in response to different concentrations of Hcy (0.0-100.0 μM).

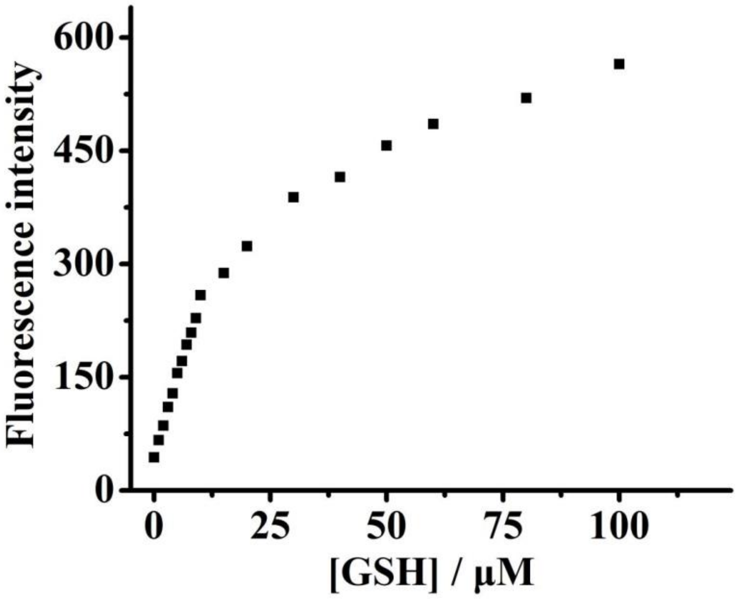

Figure S4. Fluorescence intensity of probe **1** (10.0 μM). at 670 nm in response to different concentrations of GSH (0.0-100.0 μM).

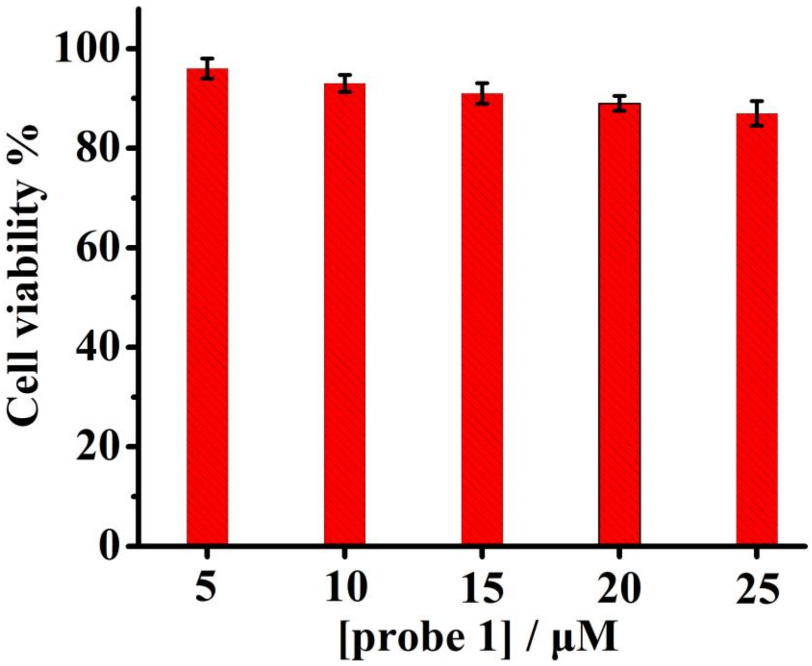

Figure S5. Cell viability of MCF-7 cells after treatment with indicated concentrations of probe **1** after 24 hours.

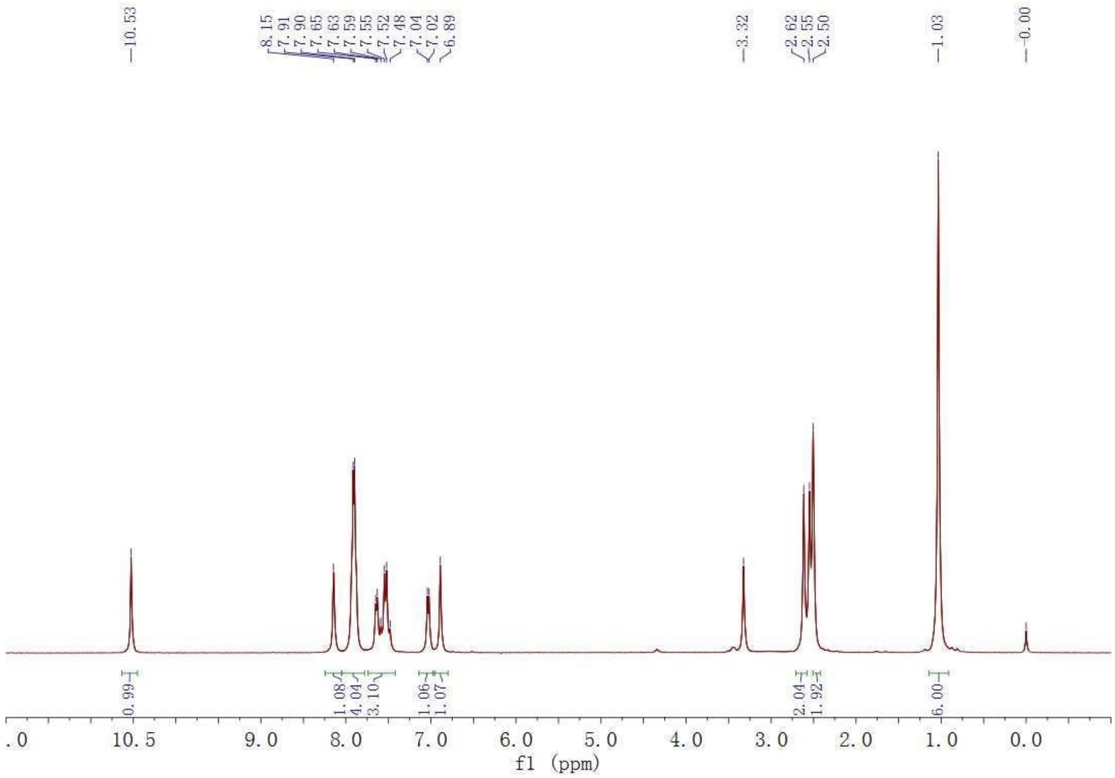

Figure S6. ^{1}H NMR spectrum of dye **2** in DMSO-d_6.

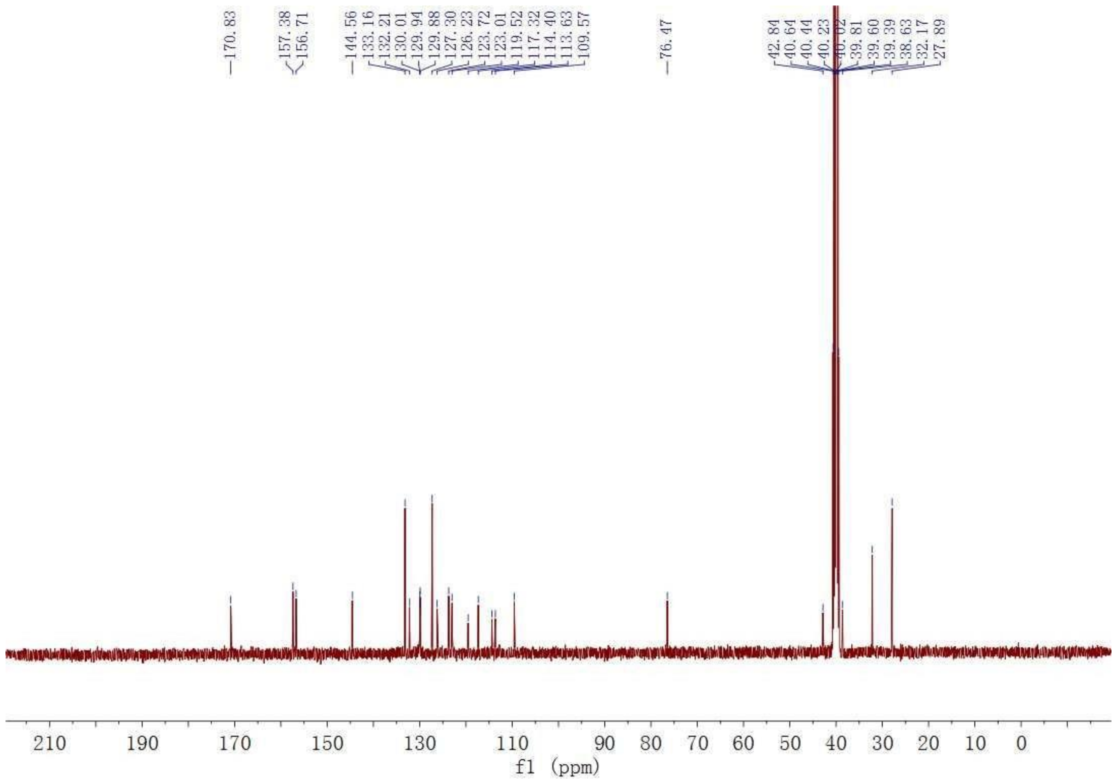

Figure S7. ^{13}C NMR spectrum of dye **2** in DMSO-d_6.

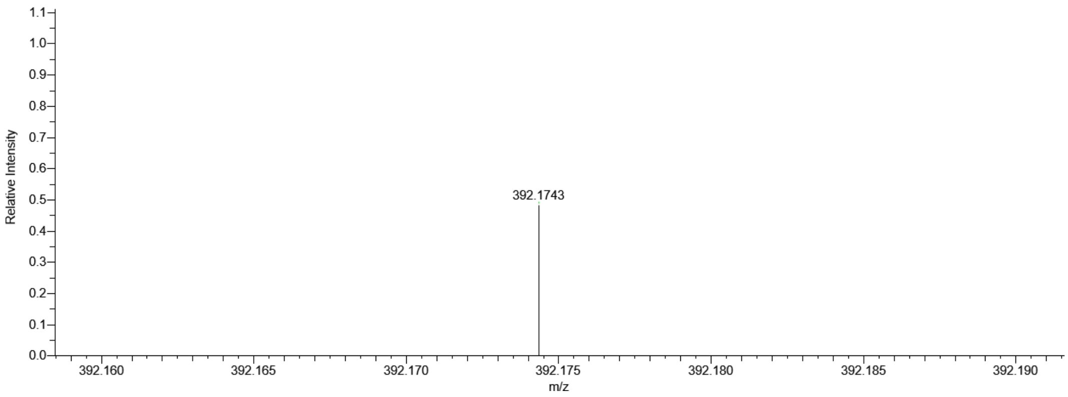

Figure S8. Mass spectrum of dye **2**.

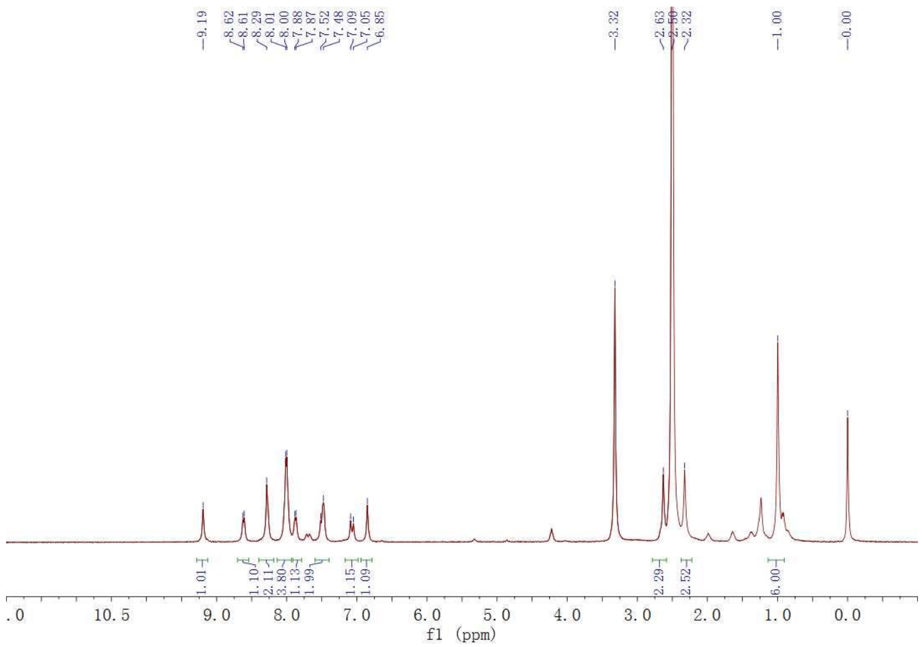

Figure S9. ^{1}H NMR spectrum of probe **1** in DMSO-d_6.

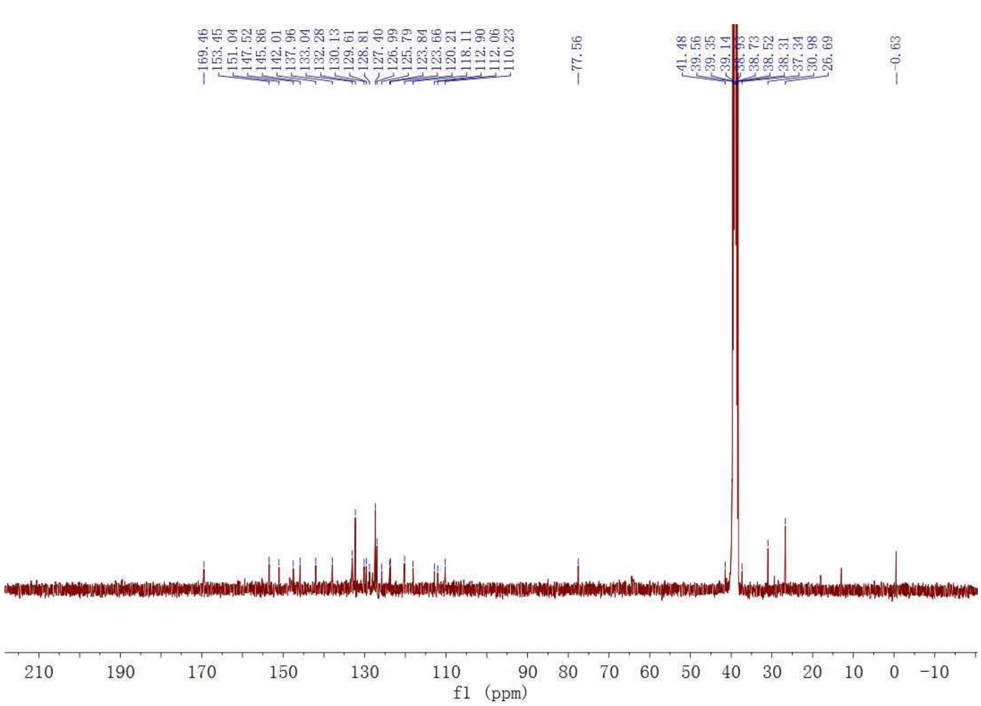

Figure S10. ^{13}C NMR spectrum of probe **1** in DMSO-d_6.

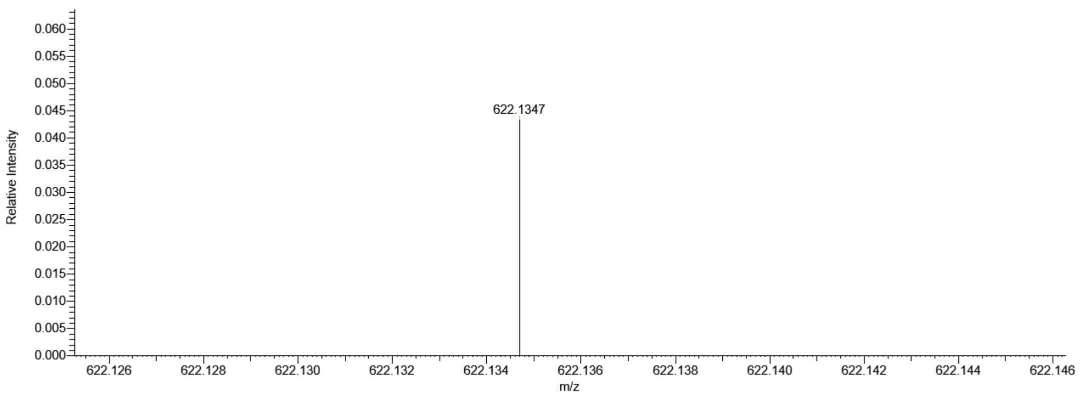

Figure S11. Mass spectrum of probe **1**.

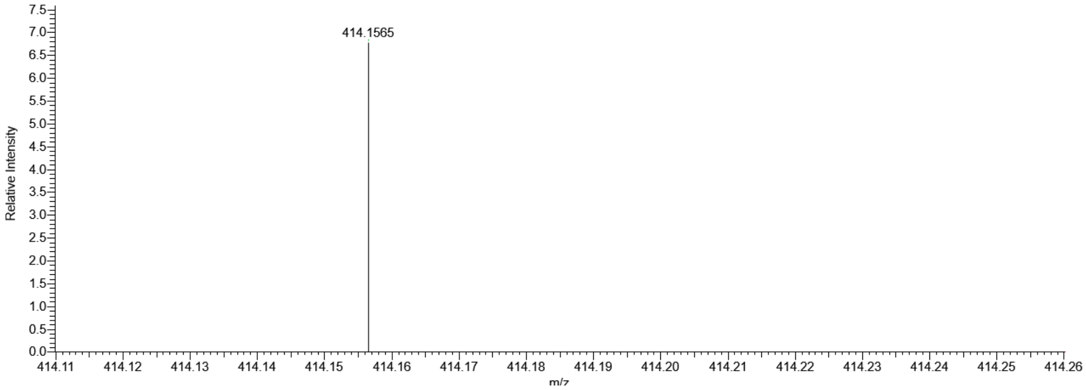

Figure S12. Mass spectrum of probe **1** with Cys.
